# Development of AI competencies within the medical curriculum

**DOI:** 10.3389/fmed.2026.1899639

**Published:** 2026-08-21

**Authors:** María Belén Morales-Cevallos, Diego Fabián Vique López, Catherine Hortensia Martínez Avalos, Brigette Carolina Huaraca Morocho, Jhonatan David Silva Ameza, Marwin Leandro Lavayen Leon

**Affiliations:** 1 Universidad Católica Santiago de Guayaquil, Guayaquil, Ecuador; 2 Italian University Institute of Rosario (IUNIR), Rosario, Argentina; 3 Facultad de Salud Pública, Escuela Superior Politécnica de Chimborazo (ESPOCH), Riobamba, Ecuador; 4 Ministerio de Salud Pública del Ecuador, Morona Santiago, Ecuador; 5 Universidad Ecotec, Samborondón, Ecuador

**Keywords:** AI, clinical education, competencies, curriculum integration, digital health, education, medical curriculum, review

## Abstract

**Introduction:**

The rapid integration of artificial intelligence (AI) into healthcare is transforming clinical practice and redefining the competencies required of future physicians. As AI increasingly supports diagnostics, clinical decision-making, and healthcare management, medical curricula must evolve to ensure graduates possess the knowledge, skills, and ethical competencies necessary to interact effectively with AI-enabled systems.

**Methodology:**

A systematic review was conducted following PRISMA guidelines. Literature searches were performed in PubMed, Scopus, and IEEE Xplore for studies published between 2020 and 2025. Eligibility criteria focused on original studies addressing AI competency development, curricular interventions, educational frameworks, and AI-related training within medical education. Following screening and eligibility assessment, 20 studies were included in the final synthesis.

**Results:**

AI literacy was the most frequently identified competency, alongside clinical AI applications, data science, ethical reasoning, critical appraisal, and human–AI collaboration. Integrated and longitudinal curriculum models emerged as the predominant approaches for competency development. Active learning strategies, particularly simulations, workshops, project-based learning, and authentic clinical applications, were consistently associated with positive educational outcomes.

**Discussion:**

The evidence indicates a transition from isolated AI educational initiatives toward competency-based and longitudinal curriculum integration. Effective AI education extends beyond technical literacy and increasingly incorporates ethical, clinical, and professional competencies necessary for responsible AI adoption in healthcare.

**Conclusion:**

AI competencies should be recognized as a core component of the medical curriculum. Integrated, longitudinal, and active learning-based educational models provide the strongest foundation for preparing future physicians to critically evaluate, ethically govern, and effectively collaborate with AI technologies in clinical practice.

## 1 Introduction

The integration of artificial intelligence (AI) into the healthcare landscape represents one of the most significant technological inflections in the history of modern medicine (1), yet the most critical challenge lies not in the algorithms themselves (2), but in the structural relationship between these technologies and the medical curriculum (3). As deep learning models and predictive analytics transition from experimental frameworks to essential bedside decision-support tools (4), the medical profession faces a mandate to evolve the physician’s role from a primary information synthesizer to an augmented practitioner (5). This evolution is fundamentally predicated on the systematic development of AI competencies (6), which must be woven into the very fabric of medical training rather than treated as an elective afterthought (7). Despite the rapid acceleration of AI capabilities in clinical settings (8), a profound competency gap remains, highlighting a disconnect between the pace of technological adoption and the agility of educational reform (9).

This educational gap is largely a product of the historical rigidity of the traditional Flexnerian model (10), which has prioritized a deep foundation in biological sciences for over a century. While this model has successfully produced generations of clinicians (11), it is currently under immense strain as it attempts to accommodate the exponential growth of digital health data. The relationship between AI and the curriculum is often characterized by a “siloed” approach, where computational literacy is isolated from clinical reasoning (12). However, for AI to be truly effective (13), it must be integrated as a cross-disciplinary pillar that touches every stage of training, from pre-clinical biosciences to graduate medical education. The sheer volume of medical knowledge is now estimated to double every few months (14), making the traditional rote-memorization model unsustainable and positioning AI as a necessary cognitive prosthetic that students must learn to manage (15).

Defining the relationship between AI and the medical curriculum requires a departure from viewing technology as a mere tool (16). Instead, it must be viewed as a new fundamental science, akin to anatomy or pharmacology (17). AI fluency involves a multidimensional competency set that encompasses technical literacy (18), data stewardship, and the critical appraisal of machine learning outputs within the context of patient care. Technical literacy in this domain does not require every physician to become a computer scientist (19), but it does necessitate a functional understanding of how supervised and reinforcement learning models arrive at their conclusions. Without this foundational knowledge being formally integrated into the curriculum (20), clinicians cannot adequately navigate the “black-box” nature of the systems they are asked to use, leading to a precarious reliance on proprietary technologies without professional oversight (21).

The ethical and socio-technical dimensions of AI further complicate its relationship with medical education (22). AI algorithms are known to perpetuate systemic biases if training data lacks diversity (23), yet current curricula rarely provide students with the framework to identify or mitigate these digital health inequities. The relationship here is one of professional responsibility; the physician must act as the ultimate arbiter of algorithmic output (24). To do so, the curriculum must foster a “human-in-the-loop” philosophy (25), teaching students to maintain clinical intuition while leveraging machine precision. This prevents the psychological trap of automation bias, where a student or resident might over-rely on a computer-generated diagnosis even when it contradicts physical findings (26).

Current literature reveals a fragmented landscape regarding the actual implementation of these educational goals (27). While some institutions have pioneered “AI tracks,” these are often segregated from the core curriculum, creating a disparity in AI readiness among graduates (28). The relationship between institutional policy and curricular change is often hindered by the saturated nature of medical school schedules (29). Educators face the significant challenge of introducing AI competencies without displacing essential clinical skills (30). Some advocate for longitudinal integration, where AI concepts are incorporated into existing courses that contribute to the medical curriculum (31), while others suggest a modular approach. The lack of a global consensus on this relationship means that the “AI-literate physician” remains an aspiration rather than a standard output of medical training (32).

Furthermore, the rationale for this systematic review is grounded in the need for a standardized roadmap that defines how AI should relate to established medical disciplines (33). As accrediting bodies begin to evaluate technological literacy, a synthesis of existing pedagogical interventions is essential to move beyond theoretical discussions (34). We must identify which teaching methodologies—be it project-based learning or interdisciplinary simulation—best foster the specific competencies required for modern practice (35). The relationship between faculty development and student learning is also a critical hurdle; many educators lack the training to lead these new modules, necessitating a “train the trainers” approach to ensure institutional sustainability (36).

The objective of this systematic review is to analyze the current state of AI competency development by focusing specifically on how these new skills interface with existing curricular structures (37). By aggregating international research, we aim to answer questions regarding the core competencies required, the most effective delivery models, and the structural barriers that prevent AI from becoming a core component of medical education (38). We explore the intersection of medical ethics and data science to provide a clear picture of an “AI-ready” curriculum (39). This synthesis serves as a resource for curriculum deans and researchers, providing the evidence needed to transition from a 20th-century educational model to one that reflects the digital reality of 21st-century medicine (40).

The urgency of reforming the relationship between AI and the medical curriculum is driven by a commitment to patient safety and professional excellence (41). As AI becomes deeply embedded in diagnostic and therapeutic pathways, the medical community must ensure that the next generation of doctors is prepared to lead this transformation (42). This review seeks to bridge the gap between technological advancement and educational practice, offering a rigorous evaluation of the efforts made thus far. By establishing evidence-based recommendations, we hope to facilitate a more uniform adoption of AI education, ensuring that the benefits of machine intelligence are realized equitably. The path forward requires a holistic reimagining of the physician’s identity, moving toward a collaborative model where human empathy and algorithmic intelligence work in tandem to improve global health outcomes. Through this inquiry, we provide the necessary evidence to support this vital and inevitable evolution in the training of the modern physician.

## 2 Methodology

This Systematic review adhered to the PRISMA guidelines to ensure transparency and reproducibility in the identification, screening, eligibility assessment, and synthesis of the literature (43, 44). Unlike traditional systematic reviews that often aim to determine the efficacy of specific interventions, this scoping review was designed to map the current state of knowledge, identify research gaps, and clarify concepts regarding the integration of AI into curriculum medical education. The systematic literature search was executed across three major interdisciplinary databases: PubMed, Scopus, and IEEE Xplore. The temporal scope was defined as the 5 years from 2020 to 2025, a timeframe that encompasses the rapid acceleration of AI integration in medical training following the emergence of advanced machine learning and generative AI architectures. The search strategy employed a combination of Boolean operators and controlled vocabulary focused on three pillars: AI-related technologies, the field of medical curriculum.

To ensure a comprehensive analysis, this review synthesized evidence from diverse student populations, including undergraduate medical students, postgraduate residents, practicing physicians, faculty, and medical educators, as well as from different types of curricular initiatives and educational interventions. By integrating findings from these diverse sources, the review sought to develop a structured overview of the competencies, curricular models, and pedagogical strategies currently used to incorporate artificial intelligence into medical education. The analytical approach categorized the literature according to key competency and educational outcome domains, including AI literacy, ethical reasoning, critical appraisal, clinical decision-making, human-AI collaboration, student engagement, and technical skills. This synthesis illustrates how AI-related content is being incorporated into medical curricula through courses, modules, workshops, simulations, longitudinal integration, and learning activities.

### 2.1 Design and application of the PICO framework

To ensure the precision and clinical relevance of this review, we utilized a modified PICO framework (Population, Intervention, Comparison, and Outcome) (45, 46), visible in Table 1, tailored for educational research. This framework facilitated the systematic definition of the research question: *“What are the current pedagogical strategies, competency frameworks, and educational outcomes reported in the literature regarding the integration of Artificial Intelligence (AI) into the medical curriculum?”*

**Table 1:** PICO framework defining the core elements used to structure the research question and inclusion criteria for the systematic review on artificial intelligence in medical curriculum.

	Element	Description
P	Population	Undergraduate medical students, postgraduate trainees, and medical educators within academic institutions.
i	Intervention	Curricular interventions, pedagogical strategies, or formal training modules designed to develop AI-related competencies.
C	Comparison	Traditional medical curricula without explicit AI content, or alternative educational methodologies (e.g., didactic vs. competency-based).
O	Outcome	Attainment of AI competencies, including technical literacy, ethical awareness, diagnostic reasoning, and application in clinical practice.

Subsequently, a structured Boolean search strategy was applied to retrieve studies located at the explicit intersection of artificial intelligence and medical curriculum. Search terms were selected to identify studies involving medical curriculum, AI-related educational interventions, and teaching or training contexts relevant to the review question. The search strategy aimed to identify studies that met predefined eligibility criteria for the period 2020–2025. The search formulation used in the selected databases is summarized in Table 2.

**Table 2:** Search strategy and number of records retrieved by database for publications on AI competencies in medical education (2020–2025).

Database	Query	Records
Scopus	(“AI”) AND (“Medical”) AND (“Education”) AND (“Curriculum”) AND (“Competencies”)	95
PubMed		239
IEEE Xplore		12

### 2.2 Database selection and search strategy

This review searched Scopus, PubMed, and IEEE Xplore to identify publications explicitly indexed at the intersection of artificial intelligence, medical education, and curriculum development. These databases were selected to cover the biomedical, educational, and technical dimensions of the topic. PubMed was used to capture literature in clinical education. Scopus was included for its broad interdisciplinary coverage across health sciences, educational theory, and applied research. IEEE Xplore was included to identify technically oriented publications with potential relevance to medical training.

The search was limited to peer-reviewed publications published between 2020 and 2025. This predefined time window was selected to capture the recent phase of AI adoption in medical training, including studies addressing AI literacy, educational applications of machine learning, and emerging teaching-oriented uses of generative AI. The Boolean search strategy was intentionally specific and designed to prioritize publications in which the educational focus was explicit in the indexed record. The core search structure combined terms related to artificial intelligence, medical education, curricula, and competencies. This approach was selected to reduce retrieval of technically focused studies in clinical settings that did not include a teaching, training, or curriculum-related objective. The search formulation applied in each database is summarized in Table 2.

The same core search structure was applied across databases, with syntax adapted where required by each platform. This focused strategy was intended to identify publications explicitly indexed at the intersection of artificial intelligence and medical curricula. The selection of specific databases was driven by their unique contributions to the research scope:

- **Scopus** was selected for its robust interdisciplinary coverage, encompassing health sciences, educational theory, and broader applied research.
- **PubMed** served as a foundational resource for retrieving highly specific biomedical and medical education literature.
- **IEEE Xplore** was included to ensure the capture of highly technical and AI-oriented educational applications that might be less prominent in clinical-only databases.

Because the literature on artificial intelligence in healthcare is broad, this strategy prioritized publications with a clear pedagogical, curricular, or learner-related focus and reduced inclusion of AI clinical studies without an educational objective. This search design should be interpreted as a focused strategy for a structured educational synthesis, not as an exhaustive search of all AI-related clinical literature.

**Figure 1:**
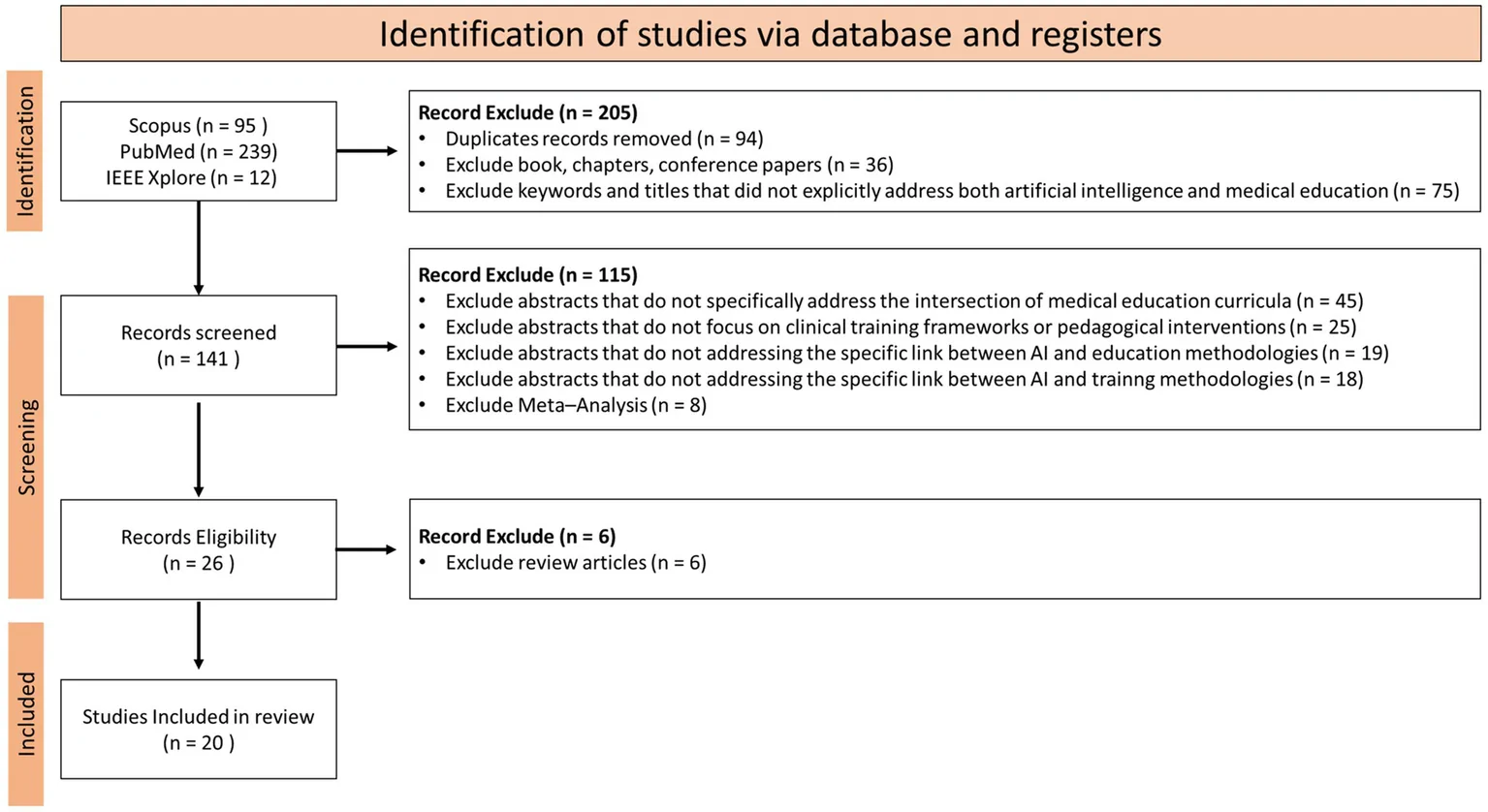
PRISMA 2020 flow diagram showing the identification, screening, eligibility assessment, and inclusion of publications in the review.

The final search was conducted on May 27, 2026. Specific search strategies were developed for PubMed, Scopus, and IEEE Xplore, using combinations of controlled vocabulary, when available, and free-text terms related to artificial intelligence, medical education, curriculum, and competencies. The search syntax was adapted to the indexing structure of each database. Searches were limited to peer-reviewed publications published between January 2020 and December 2025. The complete search strings, search fields, filters applied, and the number of records retrieved from each database are reported in Figure 1 and Table 2. Retrieved records were identified and duplicates removed before filtering using an internal algorithm employed by the authors.

**Figure 2:**
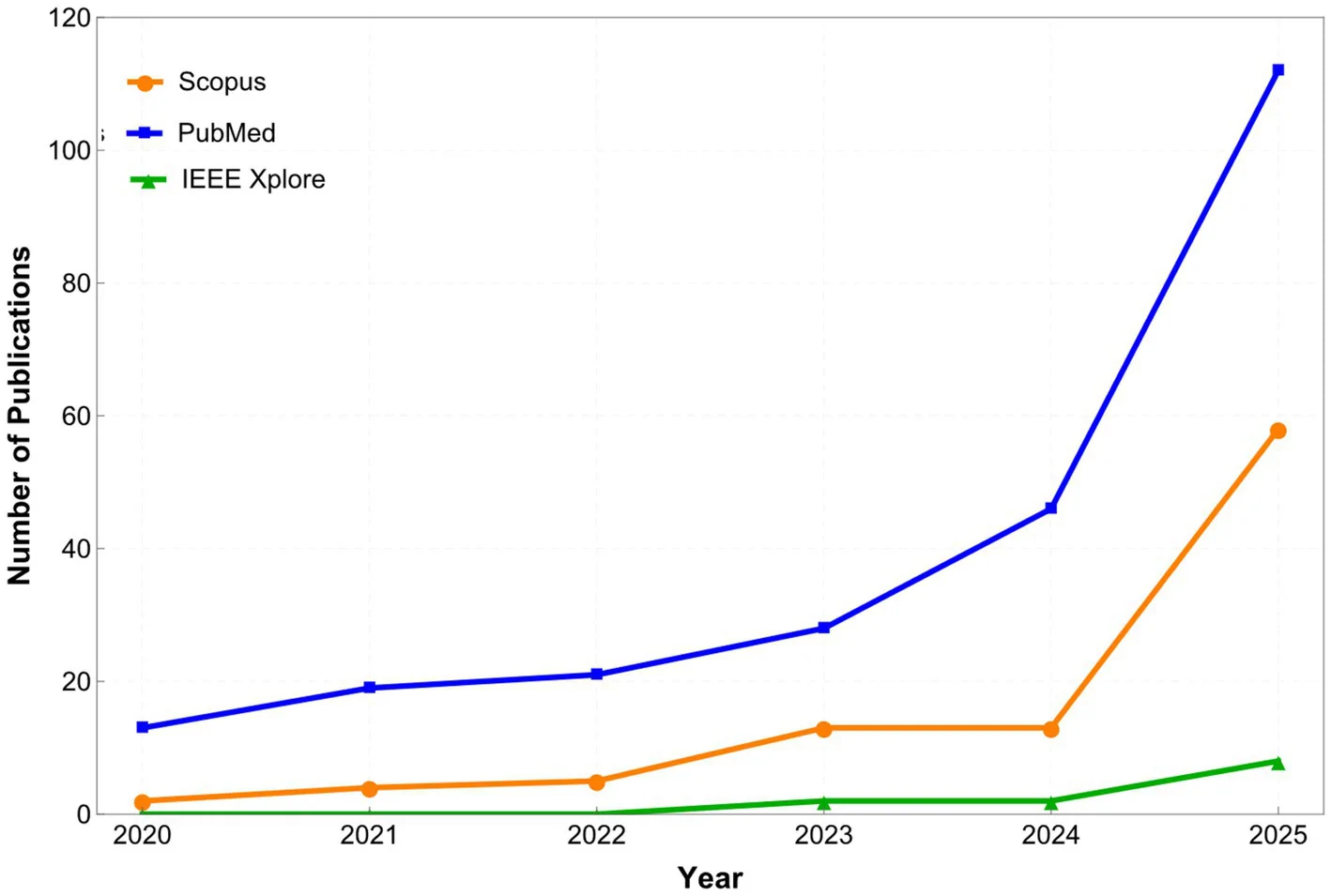
Annual number of publications indexed in Scopus, PubMed, and IEEE Xplore from 2020 to 2025 related to artificial intelligence in medical curriculum.

The volume of records retrieved demonstrated a consistent upward trend across all three databases throughout the study period, reflecting the growing academic interest in integrating artificial intelligence into medical training. Scopus yielded the highest cumulative number of records, showing a notable increase from 2 publications in 2020 to 58 by 2025. During this same timeframe, records from PubMed rose from 13 to 112, while publications in IEEE Xplore, which remained absent in the initial years, reached 8 by 2025. These results, which characterize the annual distribution of records retrieved by the search strategy, are visually summarized in Figure 2.

### 2.3 Identification

The record identification process was structured according to PRISMA. Figure 1 summarizes the stages of record identification, screening, eligibility assessment, and final inclusion.

The identification phase began with a comprehensive search across three databases, retrieving a total of 346 records: 95 from Scopus, 239 from PubMed, and 12 from IEEE Xplore. From these initial results, 205 records were excluded. Specifically, 94 were duplicates, 36 were identified as books, chapters, or conference papers, and 75 were excluded because their keywords and titles did not explicitly address both artificial intelligence and medical education. This resulted in 141 records proceeding to the screening phase.

### 2.4 Screening

During the screening phase, 115 records were excluded based on abstract assessment for failing to address core thematic areas: medical education curricula (*n* = 45), clinical training frameworks or pedagogical interventions (*n* = 25), links between AI and education methodologies (*n* = 19), links between AI and training methodologies (*n* = 18), and meta-analyses (*n* = 8). Following this stage, 26 records were assessed for eligibility.

### 2.5 Eligibility

The 26 records remaining after the screening process underwent a detailed full-text examination to determine their final relevance to the review question. During this stage, investigators assessed each article for explicit alignment with the core elements of the study: artificial intelligence and medical education. Publications that focused exclusively on technical diagnostic model performance or clinical workflow automation without an educational objective were removed.

### 2.6 Inclusion

During this stage, 6 review articles were excluded from the primary analytical dataset. These review articles were retained to inform the discussion section as a basis for contextual comparison. Following this final assessment, 20 original publications were selected and retained for the review.

## 3 Results

The final analysis focused on 20 publications addressing artificial intelligence within the domain of the medical curriculum. This sample reflects a growing body of literature examining AI-related educational contexts, ranging from foundational AI literacy initiatives and curricular proposals to technology-supported training approaches. The selected publications encompass diverse environments, including undergraduate medical education, postgraduate training, and continuing professional development.

### 3.1 AI competency domains and curricular approaches in medical education

The included studies demonstrated considerable diversity in the development and implementation of AI competencies across medical education programs, encompassing undergraduate students, postgraduate trainees, and faculty members (Table 3). The evidence indicates that AI competency development extends beyond technical knowledge acquisition to include critical appraisal, ethical reasoning, human–AI interaction, and clinical decision-making competencies. Across the reviewed literature, curricular initiatives were designed to address the growing need for healthcare professionals capable of understanding, evaluating, and responsibly applying AI technologies in clinical practice (47–66).

**Table 3:** Characteristics of AI competency domains, curricular approaches, faculty roles, learner engagement, and ethical considerations in medical education.

Reference	Learner group	Curriculum focus	AI competency domain	Curricular format	Faculty role	Learner role	Equity/Ethics considerations
AlZaabi et al. (47)	Undergraduate	AI literacy / Clinical AI / Ethics / Decision support	Knowledge / Skills / Attitudes / Ethics / Critical appraisal	Course / Module	Instructor / Facilitator / Evaluator	Active	Bias / transparency / accountability / patient safety
Kumaresan et al. (48)	Undergraduate	AI literacy / GenAI	Skills / Critical appraisal	Online	Facilitator	Active	Bias / privacy / transparency
Campbell et al. (49)	Undergraduate	Clinical AI / Decision support	Skills / Critical appraisal / Human-AI interaction	Simulation / Clerkship	Evaluator / Co-designer	Evaluator of AI output	Transparency / accountability / patient safety
Höhne et al. (50)	Undergraduate	Clinical AI / Decision support	Skills / Human-AI interaction	Course / Simulation	Instructor / Facilitator / Evaluator	Active	Patient safety / transparency
Yazdi et al. (51)	Undergraduate / Faculty	AI literacy / Ethics	Knowledge / Attitudes / Ethics	Course / Module	Instructor / Evaluator	Passive	Bias / privacy / ethics
Kim et al. (52)	Undergraduate / Faculty	AI literacy / Ethics / Clinical AI	Knowledge / Skills / Ethics / Critical appraisal	Curriculum / Module	Co-designer / Facilitator	Active	Ethics / legal responsibility / transparency / accountability
Gale et al. (53)	Postgraduate / Faculty	AI literacy / Data science	Attitudes / Human-AI interaction	Module	Instructor / Facilitator	Active	Bias / transparency / accountability / patient safety
McNulty et al. (54)	Undergraduate / Graduate / Postgraduate / Faculty	AI literacy / Data science / Clinical AI	Knowledge / Skills	Course / Module	Instructor / Facilitator / Evaluator	Active	Bias / transparency / accountability / patient safety
Waibel et al. (55)	Undergraduate	AI literacy / Data science / Ethics / Clinical AI	Knowledge / Skills / Ethics	Course / Module	Instructor / Facilitator	Active	Bias / privacy / transparency / patient safety
Nikhil et al. (56)	Undergraduate / Postgraduate / Faculty	AI literacy / Clinical AI / Ethics	Knowledge / Attitudes / Human-AI interaction	Online	Evaluator	Passive	Bias / privacy / accountability / patient safety
Sallam et al. (57)	Undergraduate	GenAI / Ethics / AI literacy	Attitudes / Ethics / Human-AI interaction	Module	Facilitator / Evaluator	Reflective learner	Bias / privacy / transparency / inequalities in care
Moldt et al. (58)	Undergraduate / Faculty	AI literacy / Data science / Ethics / Clinical AI	Knowledge / Skills / Ethics / Critical appraisal	Curriculum / Module	Co-designer / Facilitator	Active / Co-creator	Bias / transparency / privacy / accountability
Krive et al. (59)	Undergraduate	AI literacy / Clinical AI / Data science / Ethics / Decision support	Knowledge / Skills / Attitudes / Critical appraisal / Human-AI interaction	Course / Online	Instructor / Facilitator / Evaluator / Co-designer	Active / Co-creator	Bias / transparency / patient safety / accountability
Weidener et al. (60)	Faculty / Undergraduate	AI literacy / Ethics / Data science / Clinical AI	Knowledge / Skills / Ethics / Critical appraisal / Human-AI interaction	Curriculum / Module	Co-designer / Facilitator	Active	Bias / privacy / transparency / accountability / patient safety
Moldt et al. (61)	Undergraduate	AI literacy / GenAI / Ethics	Knowledge / Attitudes / Ethics / Human-AI interaction	Course / Online	Instructor / Facilitator	Active	Privacy / transparency / patient safety / human contact concerns
Shankar et al. (62)	Faculty	AI literacy / Decision support	Knowledge / Skills	Curriculum	Co-designer / Evaluator	Active	Transparency / accountability / patient safety
Hu et al. (63)	Postgraduate	AI literacy / Clinical AI / Data science	Knowledge / Skills / Critical appraisal	Workshop	Instructor / Facilitator / Evaluator	Active	Bias / transparency / patient safety
Shimizu et al. (64)	Undergraduate / Faculty	GenAI / AI literacy / Ethics	Knowledge / Skills / Ethics / Critical appraisal	Curriculum / Workshop	Co-designer / Facilitator	Active / Co-creator	Bias / privacy / transparency / accountability / patient safety
Fazlollahi et al. (65)	Undergraduate	Clinical AI / Decision support	Skills / Human-AI interaction / Critical appraisal	Simulation	Evaluator / Facilitator	Active	Transparency / accountability / patient safety
Karaca et al. (66)	Undergraduate	AI literacy / Ethics / Data science	Knowledge / Skills / Attitudes / Ethics	Module	Evaluator / Curriculum developer	Passive	Ethics / medico-legal issues / accountability

**Figure 3:**
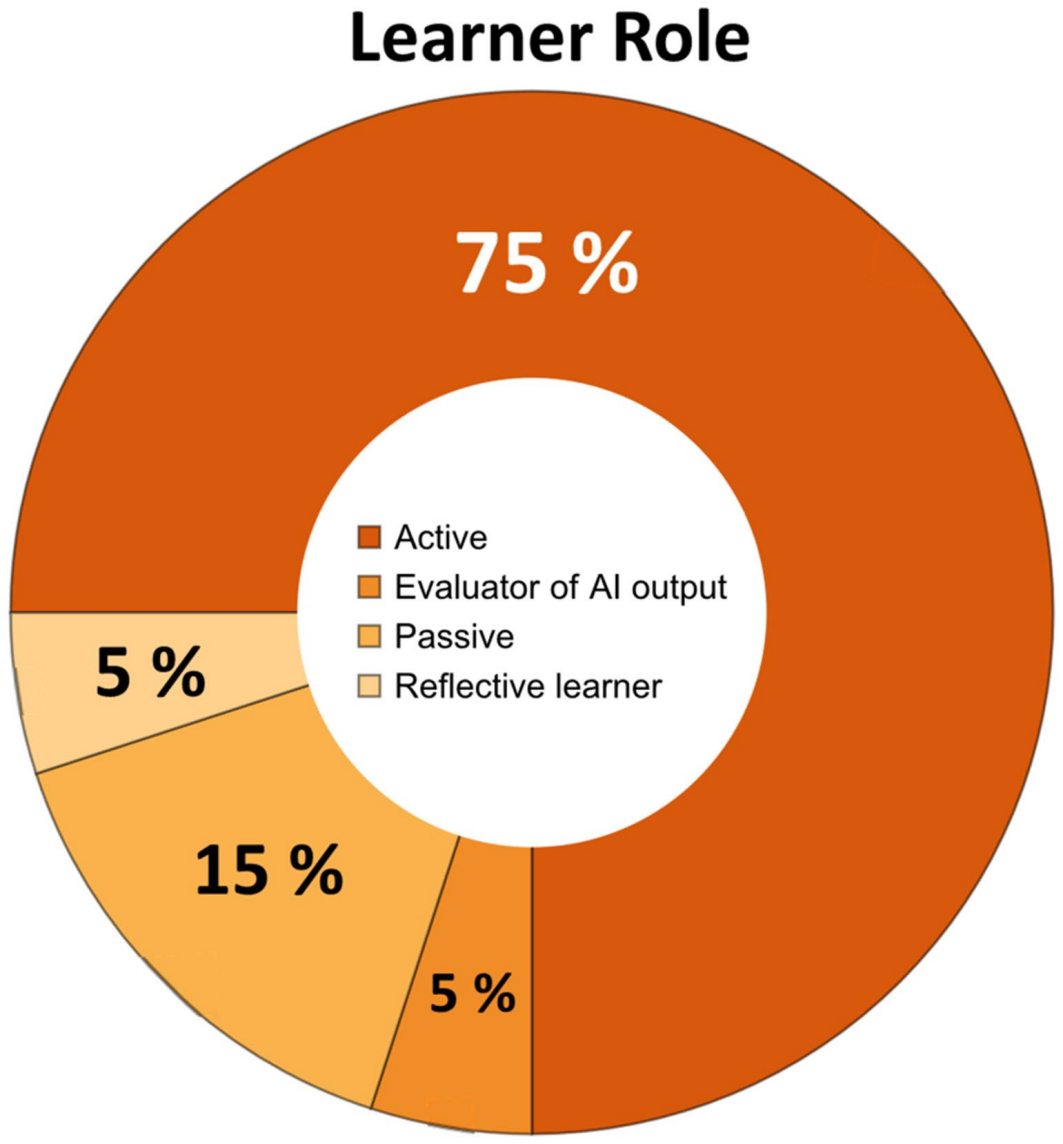
Distribution of learner roles within AI competency development initiatives in medical education, demonstrating the predominance of active learner engagement, followed by passive learners, evaluators of AI outputs, and reflective learners.

As illustrated in Figure 3, learner engagement was predominantly characterized by active participation, accounting for 75% of the identified learner roles. Passive learner roles represented 15% of the studies, while evaluators of AI outputs and reflective learners each accounted for 5%. This distribution reflects a clear shift toward learner-centered educational approaches that emphasize experiential learning, critical thinking, and active engagement with AI technologies. The predominance of active learner participation suggests that contemporary AI curricula increasingly prioritize practical application and collaborative learning rather than traditional didactic instruction alone.

AI literacy emerged as the most frequently reported curricular focus, appearing across the majority of studies (47, 48, 51–56, 58–64, 66). These initiatives sought to provide learners with foundational knowledge regarding AI principles, machine learning algorithms, data science concepts, and the potential applications of AI in healthcare. Clinical AI and decision-support systems also represented major curricular themes, particularly in studies focused on diagnostics, imaging interpretation, and clinical reasoning (47, 49, 50, 52, 54–56, 58–60, 63, 65). More recently, generative AI (GenAI) has emerged as an increasingly important area of educational focus, reflecting the rapid integration of large language models and conversational AI technologies into healthcare and medical education (48, 57, 61, 64). Data science competencies were similarly emphasized, particularly in relation to data interpretation, predictive analytics, and evidence-based decision-making (53–55, 58–60, 63, 66).

The competency domains identified across the studies encompassed multiple dimensions of AI proficiency. Knowledge and skills were the most frequently reported competencies (47, 52, 54, 55, 58–60, 62–64, 66), reflecting the importance of understanding AI concepts and applying AI tools in healthcare settings. Ethical competencies and attitudes toward AI were also widely represented (47, 51, 52, 55, 57, 58, 60, 61, 64, 66), highlighting the need for learners to critically consider issues related to professional responsibility and the societal implications of AI. Critical appraisal competencies, including the ability to evaluate AI outputs, interpret algorithmic performance, and recognize limitations, were emphasized in several studies (47–49, 52, 58–60, 63–65). Human–AI interaction competencies were similarly identified as essential for ensuring effective collaboration between healthcare professionals and AI systems while maintaining clinical judgment and patient-centered care (49, 50, 53, 56, 57, 59–61, 65).

A wide range of curricular formats was employed to facilitate competency development. Traditional courses and modules were the most commonly reported educational approaches (47, 50, 51, 54, 55, 57, 60, 66), whereas online learning environments, workshops, simulations, and clerkship experiences provided opportunities for technology-enhanced and experiential learning (48–50, 56, 59, 61, 63–65). Several studies adopted integrated curriculum models in which AI concepts were embedded throughout existing medical curricula rather than delivered as isolated educational units (52, 58, 60, 62, 64).

Faculty members fulfilled diverse educational roles, including instructors, facilitators, evaluators, curriculum developers, and co-designers of learning experiences (47, 49, 50, 52, 54, 58–60, 62–64, 66). These evolving roles reflect the interdisciplinary nature of AI education and the need for collaborative curriculum design. Also, the growing involvement of learners as co-creators of knowledge in some programs (58, 59, 64) underscores the importance of participatory educational strategies in promoting meaningful competency development.

Ethical and equity considerations were consistently incorporated across AI curricula. Common themes included algorithmic bias, transparency, accountability, privacy, legal responsibility, and patient safety (47–66). Several studies additionally highlighted concerns related to healthcare inequities, medico-legal challenges, and the preservation of human-centered care in increasingly AI-enabled healthcare environments (57, 61, 66). Overall, the findings suggest that AI competency development in medical education is evolving toward comprehensive educational frameworks that integrate technical expertise, ethical awareness, critical evaluation, and responsible clinical application.

### 3.2 Educational outcomes of AI competency development in medical education

**Figure 4:**
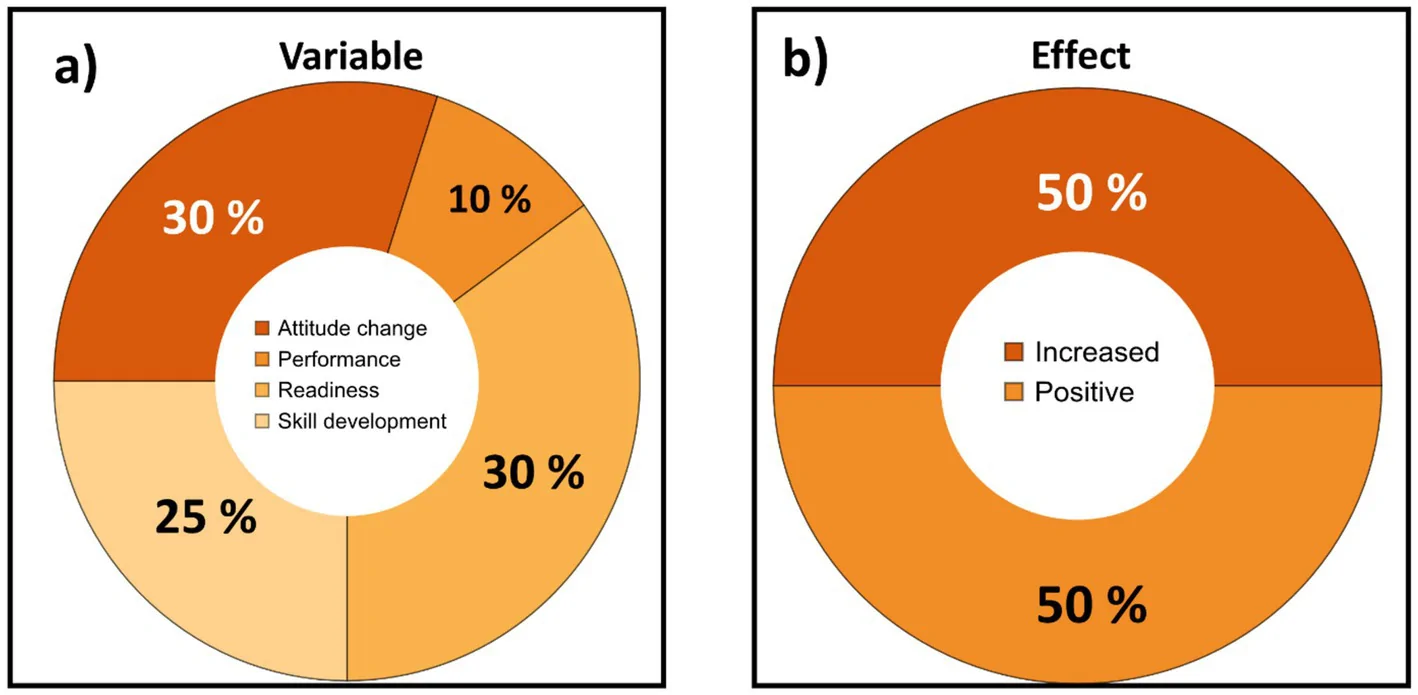
Distribution of identified pedagogical variables and observed effects in AI-integrated medical education. **(a)** Classification of core educational variables, including attitude change, performance, readiness, and skill development. **(b)** Distribution of reported effects, categorized by increased competency and positive learning outcomes.

The identified outcomes expose in Table 4, were categorized into four principal domains: readiness, attitude change, skill development, and performance. As illustrated in Figure 4a, readiness was the most frequently assessed outcome, accounting for 35% of reported variables, followed by attitude change (30%), skill development (25%), and performance (10%). This distribution indicates that current AI education initiatives primarily focus on preparing learners for future engagement with AI technologies and fostering positive perceptions toward AI integration, while comparatively fewer studies evaluate objective performance outcomes.

**Table 4:** Educational outcomes, effect directions, study characteristics, and limitations of AI competency development initiatives in medical education.

Reference	AI topic / Domain	Country	Variable	Effect	Limitations
AlZaabi et al. (47)	ML / GenAI / CDS	Oman	Readiness	Increased	Single institution, self-reported readiness, no pre-post comparison
Kumaresan et al. (48)	ML / NLP / GenAI	India	Skill development	Increased	Perception-based study, outcomes evaluated
Campbell et al. (49)	NLP / GenAI	USA	Performance	Increased	Limited to written OSCE notes, retrospective design
Höhne et al. (50)	Imaging / AI-supported ultrasound	Germany	Skill development	Increased	Small sample, pilot study, short follow-up, no real patients included
Yazdi et al. (51)	GenAI / AI applications	Iran	Readiness	Increased	Single institution, self-reported readiness, no intervention evaluated
Kim et al. (52)	GenAI / Clinical AI	South Korea	Attitude change	Increased	Perception-based study, outcomes evaluated
Gale et al. (53)	Imaging	United Kingdom	Attitude change	Positive	Perception-based study, outcomes evaluated
McNulty et al. (54)	Imaging	European Union	Skill development	Positive	Recommendations only; no implementation outcomes
Waibel et al. (55)	GenAI / Imaging	Germany	Skill development	Increased	Very small sample; single institution; self-reported outcomes
Nikhil et al. (56)	ML / Deep learning / GenAI	India	Attitude change	Positive	Self-reported data, single setting, no intervention or competency assessment
Sallam et al. (57)	GenAI / NLP	Jordan	Attitude change	Increased	Pilot study, convenience sampling, self-reported perceptions
Moldt et al. (58)	ML / Deep learning / CDS	Germany	Readiness	Positive	Qualitative single-region study; no implementation outcomes evaluated
Krive et al. (59)	ML / CDS / Analytics	USA	Skill development	Increased	Small sample, elective course, short duration, no long-term follow-up
Weidener et al. (60)	ML / CDS / Deep learning	Germany / Austria	Readiness	Positive	Qualitative design; no intervention or learner outcomes
Moldt et al. (61)	NLP / GenAI / Chatbots	Germany	Attitude change	Positive	Small sample, single-country cohort, mostly self-reported perceptions
Shankar et al. (62)	GenAI / CDS	Malaysia / Pakistan	Readiness	Positive	Faculty-only perceptions; no student outcomes; AI not directly implemented
Hu et al. (63)	ML / Deep learning / Imaging	Canada	Readiness	Increased	Small sample; single program; confidence-based outcomes
Shimizu et al. (64)	GenAI / NLP	Japan	Attitude change	Positive	Single institution; fewer students than faculty; no implementation outcomes
Fazlollahi et al. (65)	ML / Intelligent tutoring systems	Canada	Performance	Positive	Small sample, simulation-only environment, unintended negative outcomes observed
Karaca et al. (66)	ML / Deep learning / CDS	Turkey	Readiness	Positive	Self-reported readiness, conducted only in Turkish medical schools

Readiness for AI implementation emerged as the most commonly reported educational outcome. Multiple studies demonstrated increased learner preparedness, confidence, and perceived competence regarding the use of AI technologies in healthcare settings (47, 51, 63). Additional investigations reported positive readiness outcomes related to machine learning (ML), deep learning, clinical decision support (CDS), and GenAI, suggesting that educational exposure enhances learners’ awareness of AI applications and their perceived ability to engage with AI-supported clinical environments (58, 60, 62, 66). These findings were observed across undergraduate, postgraduate, and faculty populations, highlighting the broad relevance of AI readiness throughout the medical education continuum. Though, many readiness studies relied on self-reported measures, qualitative assessments, or single-institution samples, limiting the ability to draw definitive conclusions regarding actual competency acquisition (47, 51, 58, 60, 62, 66).

Attitude change represented the second most frequently reported educational outcome. Studies examining GenAI, NLP, imaging technologies, and clinical AI applications consistently reported positive or increased attitudes toward AI integration in healthcare and medical education (52, 53, 56, 57, 61, 64). Learners generally expressed optimism regarding the potential of AI to improve clinical decision-making, enhance educational experiences, and support healthcare delivery (52, 57, 61). Faculty members and healthcare professionals similarly demonstrated favorable perceptions of AI technologies and their future role in medical practice (53, 56). Nevertheless, several studies acknowledged that these findings were largely perception-based and did not directly measure competency development or behavioral change following educational interventions (52, 53, 56, 61, 64).

Skill development was another prominent outcome reported across the reviewed literature. Educational interventions involving ML, NLP, imaging technologies, AI-supported ultrasound, and clinical analytics were associated with improved technical and analytical skills (48, 50, 54, 55, 59). Learners demonstrated enhanced abilities related to data interpretation, AI-assisted clinical reasoning, diagnostic assessment, and interaction with AI-enabled tools (50, 55, 59). Some studies highlighted the value of experiential learning activities, simulations, and practical applications for strengthening competency development (48, 50, 59). Despite these positive findings, the evidence was frequently constrained by small sample sizes, short intervention periods, and reliance on self-reported evaluations rather than objective measures of skill acquisition (50, 55, 59).

Performance outcomes were less commonly evaluated but generally demonstrated favorable effects, Campbell et al. (49) reported improved educational performance associated with NLP- and GenAI-assisted grading of Objective Structured Clinical Examination (OSCE) post-encounter notes. Similarly, Rice et al. (65) found positive effects of AI-driven intelligent tutoring systems on learner performance during simulation-based surgical training. These studies suggest that AI-enhanced educational technologies may contribute to improved assessment outcomes and learning efficiency. But, both investigations were conducted in highly specific educational environments and were limited by retrospective analyses or simulation-based designs, reducing the generalizability of their findings (49, 65).

Across all AI domains—including ML, deep learning, NLP, imaging, CDS, analytics, catboats, and GenAI—the direction of effect was overwhelmingly positive or increased (47–66). Notably, no study reported a predominantly negative educational impact associated with AI competency development initiatives. Nevertheless, the quality of evidence varied considerably. Common methodological limitations included small sample sizes, single-institution settings, qualitative designs, convenience sampling, self-reported outcomes, short follow-up periods, and limited evaluation of long-term competency retention (47–66). The studies focused primarily on perceptions, recommendations, or readiness rather than objective educational outcomes (53, 54, 58, 60, 62). The findings suggest that AI competency development initiatives positively influence learner readiness, attitudes, skill acquisition, and educational performance.

Figure 4b, show AI-related educational initiatives were implemented across multiple stages of medical education, with the largest proportion occurring during the preclinical phase (50%), followed by undergraduate and clinical education (20% each), while residency and continuing medical education (CME) accounted for smaller proportions (5% each). This distribution suggests that AI competency development is primarily introduced during the early stages of medical training, with comparatively limited integration in postgraduate and continuing education settings. Readiness for AI adoption emerged as one of the most frequently reported outcomes. Several studies demonstrated increased learner preparedness to understand, evaluate, and apply AI technologies in healthcare settings (47, 51, 63). Positive perceptions of readiness were also reported in studies examining ML, deep learning, CDS, and GenAI applications (58, 60, 62, 66). These findings indicate that exposure to AI-related educational content may enhance learners’ confidence and perceived ability to engage with AI technologies in future clinical practice. Still, many readiness outcomes were based on self-reported measures and were frequently limited by single-institution designs, lack of intervention comparisons, or qualitative methodologies (47, 51, 58, 60, 62, 66).

Skill development represented another major educational outcome across the included studies. Educational interventions focusing on ML, NLP, imaging, CDS, and GenAI were consistently associated with increased or positive skill acquisition (48, 50, 54, 55, 59). Studies involving AI-supported ultrasound training, imaging interpretation, and clinical analytics demonstrated improvements in learners’ technical and analytical abilities (50, 54, 55, 59, and). Similarly, exposure to AI-driven educational tools and learning environments contributed to the development of competencies related to data interpretation, clinical decision-making, and AI-assisted problem-solving (48, 59). Despite these promising findings, several studies reported methodological limitations, including small sample sizes, short intervention durations, and reliance on self-reported outcomes rather than objective competency assessments (50, 55, 59).

Performance-related outcomes were less frequently evaluated but generally demonstrated favorable effects. Campbell et al. (49) reported improved performance associated with NLP- and GenAI-assisted assessment processes, while Rice et al. (65) found positive effects of intelligent tutoring systems on learner performance within simulation-based surgical training environments. These findings suggest that AI-enhanced educational technologies may support improved learning outcomes and assessment performance. But, both studies were conducted in highly specific contexts and were limited by retrospective or simulation-based designs, restricting the generalizability of their findings (49, 65).

Changes in learner attitudes toward AI also emerged as a recurrent outcome. Studies examining GenAI, clinical AI, imaging technologies, and NLP applications reported increased or positive attitudes regarding the role of AI in healthcare and medical education (52, 53, 56, 57, 61, 64). Learners generally perceived AI as a valuable tool for enhancing clinical practice, supporting decision-making, and improving educational experiences (52, 57, 61). Positive attitudes were also observed among faculty members and healthcare professionals, reflecting growing acceptance of AI technologies within medical training environments (53, 56). These findings were frequently based on perception-based surveys and self-reported measures, limiting conclusions regarding actual competency development or behavioral change (52, 53, 56, 57, 61, 64).

Improvements in readiness, skill acquisition, learner performance, and attitudes were consistently reported across studies examining ML, deep learning, NLP, imaging, CDS, and GenAI applications (47–66). Until now, the strength of the evidence remains constrained by methodological limitations, including small samples, single-institution settings, short follow-up periods, reliance on self-reported outcomes, and the limited availability of longitudinal evaluations.

### 3.3 Educational outcomes and evidence of AI competency development in medical education

**Figure 5:**
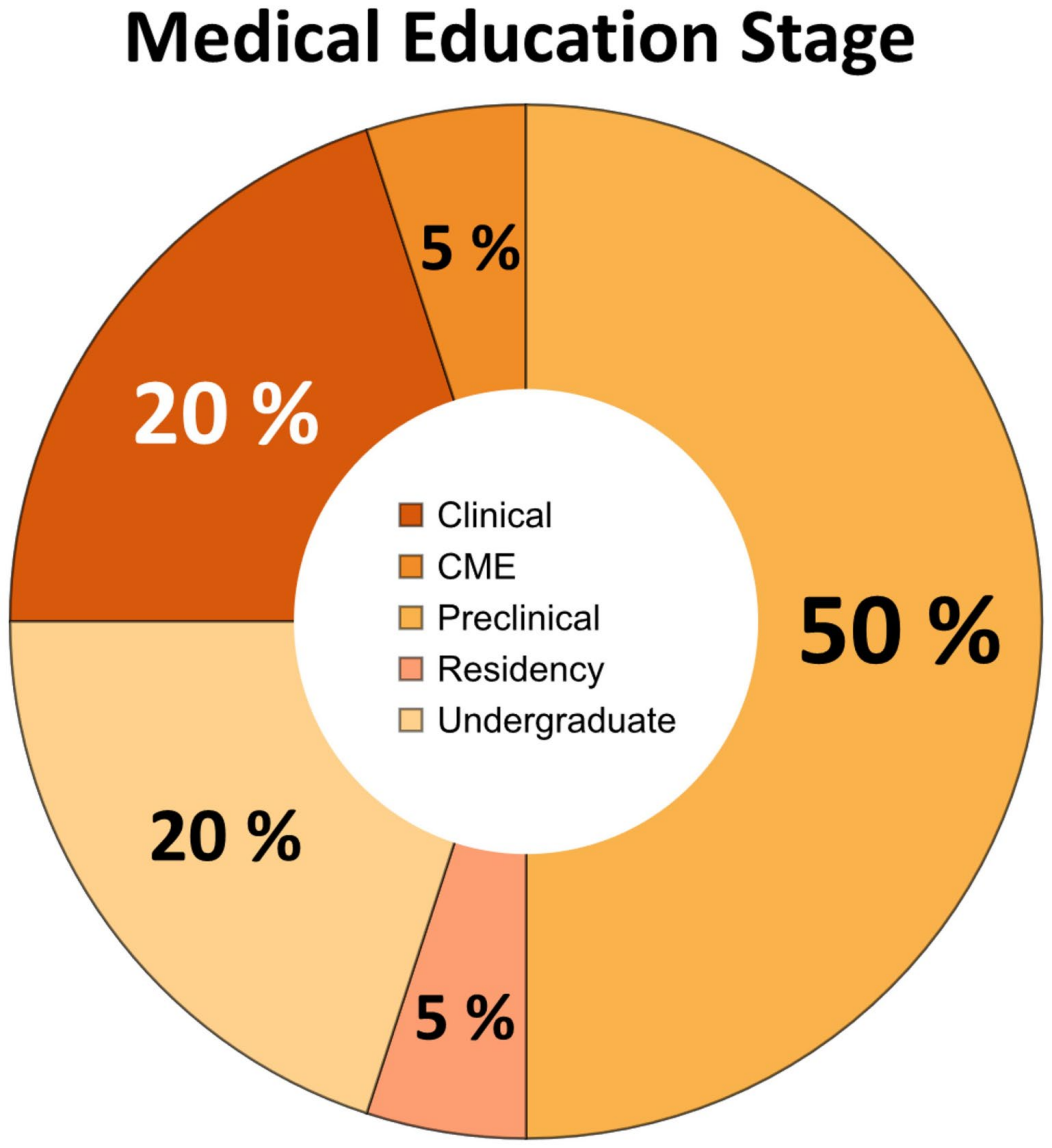
Distribution of AI competency development initiatives across stages of medical education, illustrating the predominance of preclinical implementation and the progressive integration of AI-related learning experiences throughout undergraduate, clinical, residency, and continuing medical education settings.

Table 5 shows that across the reviewed literature, AI competency development was increasingly embedded within existing curricula through integrated and longitudinal approaches rather than being delivered solely as standalone educational experiences, in Figure 5, AI-related educational initiatives were distributed across the continuum of medical education, with the greatest representation occurring during the preclinical stage (50%), followed by undergraduate and clinical education (20% each), while residency and CME accounted for smaller proportions (5%). This distribution highlights the growing emphasis on introducing AI competencies early in medical training while also recognizing the need for continued competency development throughout professional practice. Integrated curriculum models were the most frequently reported strategy for AI implementation (47, 49–51, 53, 56, 57, 60). These approaches embedded AI-related competencies within existing courses and learning experiences, allowing students to connect AI concepts with clinical reasoning, diagnostics, assessment, and healthcare delivery. Several studies advocated longitudinal integration models that progressively introduced AI concepts throughout medical training, enabling learners to develop increasingly sophisticated competencies over time (47, 52, 54, 58, 60, 62). Longitudinal approaches were particularly emphasized for fostering sustainable competency development and ensuring alignment with evolving technological advancements in healthcare.

**Table 5:** Educational characteristics, curriculum integration models, learning activities, clinical contexts, and facilitators supporting AI competency development in medical education.

Reference	Study objective	Medical education stage	Curriculum integration model	Learning activities	Clinical / Educational context	Facilitators
AlZaabi et al. (47)	Evaluate undergraduate medical students’ readiness for AI after preclinical exposure to AI-related curricular content	Preclinical / Clinical	Integrated / Longitudinal	Literature search, discussions, presentations, AI tool evaluation	Clinical reasoning / Diagnostics	Early curricular integration, interactive learning
Kumaresan et al. (48)	Evaluate effectiveness of AI-driven adaptive learning platforms on cognition, engagement, and retention	Undergraduate	Standalone	Interactive assessments, gamification, adaptive quizzes	Educational context	Real-time feedback, predictive analytics
Campbell et al. (49)	Evaluate GPT-4 automated grading system for OSCE post-encounter notes compared with human graders	Clinical	Integrated	OSCE post-encounter note grading	Clinical reasoning / Assessment	Standardized rubrics, automated feedback
Höhne et al. (50)	Compare AI-supported blended ultrasound learning versus traditional hands-on instruction for medical students	Clinical	Integrated	OSCE, ultrasound image acquisition, peer-assisted learning	Diagnostics / Radiology	Real-time AI feedback, blended e-learning, standardized app guidance
Yazdi et al. (51)	Compare AI readiness between dental faculty members and students	Preclinical / Clinical	Integrated	MAIRS questionnaire completion	Educational context	Interest in dentistry, faculty expertise
Kim et al. (52)	Explore faculty and student perceptions about integrating AI into medical curricula	Preclinical / Clinical	Longitudinal / Integrated	National online survey with free-text responses	Curriculum development / Clinical reasoning	Spiral curriculum design, hands-on practice, exposure to AI developers
Gale et al. (53)	Examine the impact of AI automation on radiography practice, workforce expertise, morale, and higher education	CME	Integrated	Critical discussion of AI automation and workforce implications	Radiology	CPD, curriculum reform, collaboration with engineers
McNulty et al. (54)	Provide recommendations for radiographer education and training across EU member states	Undergraduate / Postgraduate / CME	Longitudinal	Clinical placements, competency assessments, research training, CPD	Radiology	Standardized competency frameworks, CPD, flexible pathways
Waibel et al. (55)	Evaluate an interdisciplinary elective course on digital competencies in medicine	Clinical	Elective	Self-learning modules, practical workshops, AI image classification, telemedicine simulations, app development	Clinical reasoning / Radiology	Modular design, practical exercises, interdisciplinary teaching
Nikhil et al. (56)	Assess healthcare professionals’ knowledge, attitudes, and practices regarding AI in healthcare settings	Preclinical / Clinical / CME	Integrated	Questionnaire on AI familiarity, use, and attitudes	Clinical reasoning / Public health	High awareness of AI significance, positive perceptions of AI integration
Sallam et al. (57)	Examine fears, anxiety, mistrust, and ethical concerns of medical students toward generative AI	Preclinical / Clinical	Integrated	FAME scale survey, reflection on AI-related concerns	Clinical reasoning / Medical education	Previous exposure to genAI, curriculum adaptation
Moldt et al. (58)	Explore stakeholder perspectives and identify essential AI competencies for medical curricula	Preclinical / Clinical	Longitudinal / Integrated	Stakeholder interviews, curriculum mapping, competency identification	Clinical reasoning / Curriculum development	Stakeholder engagement, interdisciplinary collaboration, structured curriculum design
Krive et al. (59)	Develop and evaluate a competency-driven AI elective integrated into undergraduate medical education	Clinical	Elective / Integrated	Team projects, predictive analytics design, EMR quality measures, telemonitoring workflows, self-reflection	Clinical reasoning / Diagnostics / Public health	Multidisciplinary faculty, constructivist design, real-world simulations
Weidener et al. (60)	Identify essential AI-related competencies and curricular content for medical education through expert interviews	Preclinical / Clinical	Integrated / Longitudinal	Expert interviews, competency mapping	Curriculum development / Clinical reasoning	Early integration, extracurricular opportunities, longitudinal implementation
Moldt et al. (61)	Explore medical students’ attitudes and knowledge regarding AI and medical chatbots and identify curricular needs	Preclinical / Clinical	Elective / Integrated	Hybrid chatbot course, group discussions, reflective exercises, AI demonstrations	Clinical reasoning / Communication / Assessment	Interactive hybrid teaching, practical exposure, discussion-based learning
Shankar et al. (62)	Explore faculty perceptions of a flexible-length, outcomes-based undergraduate medical curriculum and the role of AI in individualized learning pathways	Undergraduate	Longitudinal / Integrated	Faculty interviews on flexible curriculum, AI-supported progression, individualized learning	Assessment / Clinical reasoning	AI-supported personalization, online learning, competency-based progression
Hu et al. (63)	Develop, implement, and evaluate a condensed AI workshop for diagnostic radiology residents	Residency	Integrated	Didactic sessions, literature case studies, programming examples	Radiology / Diagnostics	Integrated academic half-days, faculty-resident-AI engineer collaboration
Shimizu et al. (64)	Analyze the impact of generative AI on medical education curricula and identify curriculum reform strategies	Undergraduate	Integrated / Longitudinal	SWOT workshop, group discussion, curriculum strategy development	Clinical reasoning / Assessment	Faculty-student co-design, SWOT analysis, curriculum reform framework
Fazlollahi et al. (65)	Assess the pedagogical value and unintended outcomes of AI-selected technical competencies in surgical simulation training	Preclinical	Integrated	Neurosurgical VR simulation, AI-guided feedback, repeated procedural practice	Surgery / Simulation training	Personalized feedback, expert benchmarks, competency-based simulation
Karaca et al. (66)	Develop and validate a psychometric scale to assess medical students’ readiness for AI in medicine	Preclinical / Clinical	Integrated	Psychometric scale development, expert consultation, readiness assessment	Medical education / Curriculum development	Expert-informed framework, psychometric validation methods

A variety of learning activities were employed to support AI competency acquisition. Interactive educational experiences were commonly reported and included literature reviews, group discussions, presentations, AI tool evaluations, competency mapping exercises, and stakeholder interviews (47, 52, 58, 60). Technology-enhanced learning activities such as adaptive quizzes, gamification, predictive analytics exercises, chatbot demonstrations, and AI-supported simulations were also frequently utilized (48–50, 59, 61). Several studies incorporated experiential learning opportunities through clinical placements, ultrasound image acquisition, telemedicine simulations, virtual reality surgical training, and predictive analytics projects, allowing learners to apply AI concepts in authentic healthcare contexts (50, 54, 55, 59). These approaches promoted active learning and facilitated the development of practical AI competencies relevant to clinical practice.

The educational and clinical contexts in which AI competency development occurred were equally diverse. Clinical reasoning emerged as the most common application area and was integrated across multiple educational interventions (47, 49, 52, 56–60, 62). Diagnostic medicine and radiology were also prominent domains, reflecting the widespread implementation of AI technologies in imaging and diagnostic decision support (47, 50, 53–55). Additional contexts included surgery, public health, communication, assessment, and curriculum development, illustrating the broad applicability of AI across healthcare disciplines (49, 56, 58, 61). These findings suggest that AI education is increasingly moving beyond technical instruction toward context-specific applications that mirror real-world clinical environments.

Several facilitators were consistently identified as supporting successful AI competency development initiatives. Early curricular integration was frequently highlighted as a key factor promoting learner readiness and sustained engagement with AI concepts (47, 60). Interactive learning environments, practical exercises, and hands-on exposure to AI tools were also associated with positive educational experiences and improved competency development (47, 50, 55, 61). Interdisciplinary collaboration emerged as another important facilitator, with multiple studies emphasizing partnerships among clinicians, educators, engineers, computer scientists, and AI developers (52, 58, 59). In addition, competency-based progression, standardized competency frameworks, curriculum reform initiatives, and AI-supported personalized learning pathways were identified as mechanisms for strengthening AI education and promoting learner-centered development (54, 62).

Elective courses and workshops provided additional opportunities for focused competency development, particularly in undergraduate and clinical education settings (55, 59, 61). These formats allowed learners to explore advanced AI applications, participate in project-based learning, and engage in multidisciplinary problem-solving activities. Though, many authors emphasized that elective approaches should complement rather than replace broader curriculum integration efforts to ensure equitable access to AI education for all medical learners (55, 59, 61). These findings demonstrate a shift toward comprehensive educational frameworks designed to prepare future physicians for effective and ethical engagement with AI-enabled healthcare systems (47–62).

## 4 Discussion

### 4.1 Defining AI as a core component of the medical curriculum

**Figure 6:**
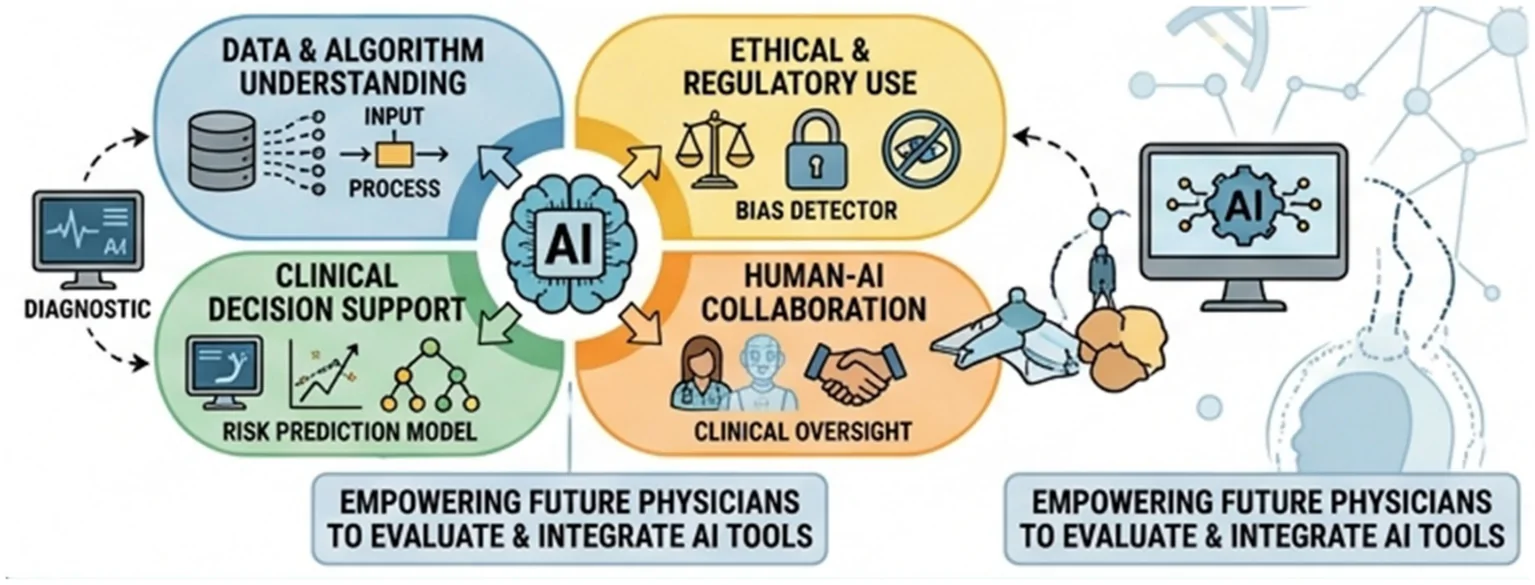
Core competency domains for integrating AI into the medical curriculum.

Medical curricula must evolve to ensure that graduates possess the competencies necessary to critically evaluate, responsibly apply, and effectively collaborate with AI systems in clinical settings. A notable finding of the reviewed literature is the predominance of AI literacy as the central curricular focus. Most studies emphasized foundational knowledge of AI principles, machine learning, data science, and emerging generative AI applications as essential learning outcomes (47, 48, 51–56, 58–64, 66). This widespread emphasis suggests growing consensus that AI literacy should be regarded as a core competency for all medical students, irrespective of future specialty choice. Similar to previous curricular transformations involving evidence-based medicine, genomics, and health informatics, AI education is progressively moving from specialized training toward a universal educational requirement. The objective is not to transform physicians into data scientists or software engineers but rather to develop sufficient understanding to interpret AI outputs, recognize limitations, and incorporate AI-assisted recommendations into clinical reasoning processes (Figure 6).

The competency domains identified in this review further support the conceptualization of AI as a core curricular pillar. Beyond technical knowledge, educational initiatives frequently addressed critical appraisal, ethical reasoning, human–AI interaction, attitudes toward technology adoption, and clinical decision support (47, 49, 50, 52, 57–60, 63–65). These findings indicate that AI competency development extends beyond learning how algorithms function. Instead, curricula increasingly seek to cultivate the ability to evaluate algorithmic performance, assess reliability and bias, understand ethical implications, and maintain professional accountability when utilizing AI-supported tools. Such competencies align closely with the broader educational objectives of medical training, including clinical judgment, patient safety, and evidence-informed decision-making.

Another important observation is the growing preference for integrated and longitudinal curricular models (47, 52, 54, 58, 60, 62, 64). Rather than introducing AI through isolated electives or standalone courses, many institutions are embedding AI-related concepts within existing educational structures. This approach facilitates contextual learning by linking AI competencies directly to clinical reasoning, diagnostics, radiology, public health, and patient care activities. Integration within established curricular frameworks may also reduce concerns regarding curriculum overcrowding while promoting progressive competency development throughout the educational continuum.

The findings also highlight that AI competency development increasingly incorporates ethical, legal, and societal dimensions (47–66). Issues such as algorithmic bias, transparency, accountability, privacy, and patient safety were consistently represented across curricular initiatives, reflecting recognition that technological proficiency alone is insufficient for responsible AI implementation. Future physicians must be equipped to navigate the complex interactions between technology, healthcare systems, and patient-centered care. AI should not be viewed solely as an emerging technology topic but as a foundational domain that intersects with clinical practice, professional ethics, and healthcare delivery.

### 4.2 Learning strategies supporting AI competency acquisition

**Figure 7:**
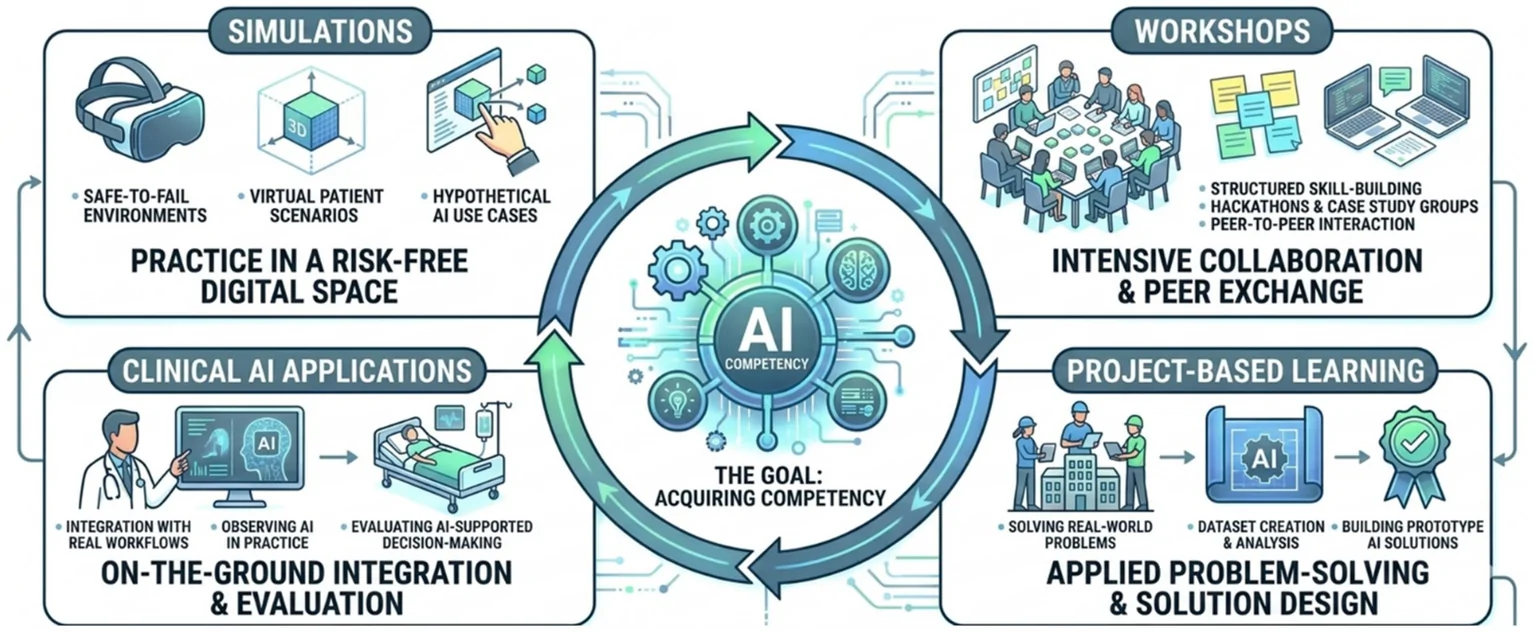
Active learning strategies supporting AI competency acquisition in medical education.

The findings of this review indicate that active learning has emerged as the dominant pedagogical approach for developing AI competencies in medical education. Across the included studies, learners were primarily engaged through interactive, experiential, and application-oriented educational activities rather than traditional lecture-based instruction alone (47–66). This trend is reflected in the predominance of active learner roles identified in the reviewed literature, where approximately three-quarters of educational initiatives required students to directly interact with AI tools, analyze outputs, participate in problem-solving activities, or engage in collaborative learning experiences. Such approaches are consistent with contemporary competency-based medical education models, which emphasize the development of practical skills, critical thinking, and professional judgment through authentic learning experiences (Figure 7).

A key advantage of active learning strategies is their ability to bridge the gap between theoretical understanding and clinical application. Several studies incorporated hands-on experiences that allowed learners to explore AI technologies within realistic healthcare contexts (47, 50, 55, 59, 61). These activities included AI tool evaluation, ultrasound image acquisition supported by AI feedback, predictive analytics projects, chatbot demonstrations, telemedicine simulations, and image classification exercises. By engaging directly with AI systems, learners developed a deeper understanding of both the capabilities and limitations of these technologies, fostering competencies that extend beyond conceptual knowledge toward practical implementation. Such experiential activities may be particularly valuable in preparing future physicians to critically assess AI-generated recommendations and appropriately integrate them into clinical decision-making processes.

Simulation-based learning represented another important educational strategy identified across the reviewed studies (49, 50, 65). Simulated clinical environments provide opportunities for learners to interact with AI-supported technologies while maintaining a safe educational setting that minimizes risks to patients. For example, AI-assisted ultrasound training, virtual reality surgical simulations, and automated assessment systems enabled learners to receive immediate feedback and repeatedly practice clinical skills (50, 65). These approaches align with established educational theories suggesting that deliberate practice and feedback are essential mechanisms for competency development. The incorporation of AI within simulation environments may further enhance learning by providing individualized guidance, performance analytics, and adaptive educational support.

Collaborative and project-based learning activities also played a significant role in AI competency acquisition (47, 52, 58, 59, 64). Several studies reported the use of group discussions, stakeholder engagement exercises, competency mapping activities, curriculum design workshops, and multidisciplinary team projects (52, 58, 64). These approaches encouraged learners to critically examine the implications of AI in healthcare while developing communication, teamwork, and problem-solving skills. Project-based experiences involving predictive analytics, electronic medical record workflows, and healthcare innovation challenges further allowed students to apply AI concepts to real-world clinical problems (59). Such activities promote deeper learning by encouraging learners to actively construct knowledge rather than passively receive information.

The increasing use of technology-enhanced learning environments also reflects the growing importance of active engagement in AI education. Adaptive learning platforms, gamified assessments, interactive quizzes, and AI-supported educational systems were associated with positive outcomes related to skill development, learner engagement, and readiness for AI implementation (48, 59, 62). These tools provide opportunities for personalized learning experiences that can accommodate varying levels of prior knowledge and learner needs. Furthermore, AI-driven educational technologies may facilitate continuous feedback and competency monitoring, supporting self-directed learning and individualized progression.

### 4.3 Comparison with prior reviews and related literature

Previous reviews have contributed significantly to defining the knowledge and skills required for future physicians. Common themes across the literature include AI literacy, machine learning fundamentals, healthcare data science, ethical and legal considerations, clinical decision support, critical appraisal of AI outputs, and responsible use of AI technologies in healthcare settings (67–72). These reviews have also emphasized the necessity of embedding AI-related competencies throughout medical education rather than limiting instruction to isolated electives or optional training opportunities (67, 69, 72). Such findings have been instrumental in shifting the discourse from whether AI should be included in medical curricula to how it should be integrated effectively (Table 6).

**Table 6:** Analysis of previous reviews contributing to the development of AI competencies within the medical curriculum.

Reference	Curriculum development contribution	Educational scope	Principal findings	Limitations	Gaps identified
Mendes et al. (67)	Proposes explicit curriculum models, integration strategies	Undergraduate, postgraduate, and continuing professional development in healthcare education	The review argues that AI should be embedded throughout medical education rather than taught as isolated content and presents the consumer–translator–developer competency model as a mechanism for differentiated learning pathways.	Limited evidence exists regarding standardized assessment frameworks and implementation across diverse institutions.	Narrative review design; lacks systematic assessment of educational outcomes and provides limited empirical evidence regarding effectiveness of proposed curriculum models. Most recommendations remain conceptual.
Kim et al. (68)	Provides comprehensive curriculum design including competencies, integration, pedagogy, assessment, faculty development, and evaluation mechanisms	Undergraduate medical education	Provides a detailed curriculum development framework consisting of 12 practical recommendations for implementing AI education within competency-based medical education	Further research is needed to determine how proposed AI competencies translate into educational and healthcare outcomes.	Recommendations are derived from literature review and expert consensus, with limited evidence regarding implementation outcomes across institutions.
Itani et al. (69)	Proposes explicit curriculum models, integration strategies	Undergraduate, postgraduate, and continuing professional development in medical education	The review highlights that AI education should prepare future physicians to critically evaluate and responsibly use AI technologies in healthcare.	Limited longitudinal studies assessing learning outcomes, and a shortage of research evaluating the effectiveness of AI ethics training on clinical practice.	Most included studies were reviews or conceptual papers, with relatively few empirical evaluations of AI educational interventions.
Lee et al. (70)	Proposes explicit curriculum models, integration strategies	Undergraduate Medical Education	Identified a strong need for systematic AI integration within undergraduate medical curricula. Proposed key curricular domains including AI fundamentals, machine learning literacy, critical appraisal of AI systems, ethical and legal implications, EHR competencies, and development of uniquely human skills such as empathy and communication.	Need for validated competency frameworks, curriculum evaluation studies, and evidence-based implementation models across medical schools	Considerable heterogeneity existed regarding curriculum content, teaching methods, competencies, and assessment strategies.
Negrete et al. (71)	Identifies competencies or educational needs but provides limited curricular structure.	Dental and Dentomaxillofacial Radiology Education	The review emphasized preparing learners to become technologically literate, ethically grounded, and clinically competent professionals capable of working alongside AI systems.	Limited evidence regarding long-term competency development, need for faculty training, curricular alignment, ethical governance, and evaluation of AI-supported learning interventions	Narrative review design with limited empirical evidence evaluating educational outcomes or curriculum effectiveness.
Tolentino et al. (72)	Proposes explicit curriculum models, integration strategies	UME, PGME, and CME	Proposed key curriculum domains including AI fundamentals, healthcare data science, strengths and limitations of AI, ethics/legal considerations, clinical applications, AI-assisted decision-making, communication of AI results, and critical appraisal skills. Emphasized that curriculum frameworks remain scarce despite growing educational activity	Need for validated curriculum frameworks, competency-based standards, longitudinal implementation models, educational theory integration, and higher-level outcome evaluations assessing behavioral and clinical impact.	Evaluation studies were limited largely to learner satisfaction and knowledge acquisition.
Our Study	Provides comprehensive curriculum design including competencies, integration, pedagogy, assessment, faculty development, and evaluation mechanisms.	Medical education continuum, preclinical, undergraduate, clinical, residency, and CME.	Provides a multidimensional synthesis of AI competency development by integrating competency domains, curriculum integration models, educational outcomes, pedagogical strategies, learner engagement, faculty roles, ethical considerations, and implementation facilitator.	The evidence remains limited by self-reported results, single-institution studies, small sample sizes, and the scarcity of longitudinal assessments provided by the studies selected in the methodology filter.	Highlights the need for standardized international competency frameworks, objective assessment tools, faculty development programs, and longitudinal evaluation of AI competency acquisition across the medical education continuum.

Despite these contributions, much of the existing literature has concentrated primarily on competency identification and curriculum recommendations. Most reviews focused on defining curricular domains, outlining educational priorities, or proposing conceptual frameworks for AI education (67–72). While these efforts provide valuable strategic direction, they offer comparatively limited evidence regarding the practical mechanisms through which competencies can be developed, reinforced, and assessed within educational environments. Furthermore, many published recommendations were derived from narrative analyses, expert opinion, stakeholder perspectives, or conceptual discussions rather than systematic examination of implemented educational interventions (67–71). This limitation has created a persistent gap between theoretical curriculum proposals and evidence-informed curriculum implementation.

An additional observation emerging from the literature is the restricted educational scope of many previous reviews. Some investigations concentrated exclusively on undergraduate medical education (68, 70), whereas others examined specialty-specific contexts such as radiology or dentomaxillofacial education (71). These focused perspectives generated important insights regarding local educational needs; however, they provided limited understanding of how AI competencies evolve across the broader continuum of medical education. Likewise, previous reviews rarely examined the relationships among curriculum integration models, pedagogical strategies, learner engagement, faculty involvement, and educational outcomes within a unified analytical framework (67–72).

The present review expands upon prior scholarship by adopting a comprehensive curriculum-centered perspective that encompasses preclinical education, undergraduate medical education, clinical training, residency programs, and continuing medical education. In contrast to earlier reviews that predominantly emphasized competency frameworks or curriculum content, this study synthesizes evidence across multiple dimensions of AI curriculum development, including competency domains, curriculum integration models, pedagogical approaches, learner roles, faculty responsibilities, ethical considerations, implementation facilitators, and educational outcomes. This multidimensional approach provides a more holistic understanding of how AI competencies are developed and sustained throughout the educational continuum.

A distinguishing contribution of the present review is its integration of curriculum structure with evidence regarding educational effectiveness. The findings reveal that integrated and longitudinal curriculum models constitute the predominant approaches to AI competency development, particularly when supported by active learning methodologies such as simulations, workshops, project-based learning, adaptive learning environments, and authentic clinical applications. In addition, the review demonstrates how these educational strategies are associated with improvements in learner readiness, skill acquisition, attitudes toward AI, and educational performance. By connecting curriculum design with measurable educational outcomes, this review advances beyond the descriptive focus that characterizes much of the previous literature.

Moreover, this review identifies implementation factors that have received comparatively limited attention in earlier syntheses, including interdisciplinary collaboration, faculty development, competency-based progression, curriculum governance, and personalized learning pathways. These elements represent critical determinants of successful curriculum adoption and sustainability, yet they have often been discussed only superficially in previous reviews (67–72). Their inclusion strengthens the practical relevance of the present findings for curriculum developers, educational leaders, and accreditation bodies seeking to operationalize AI education within existing training structures.

Taken together, the current review advances the field beyond the identification of competencies and curricular aspirations by offering an integrated framework that links what should be taught, how it should be taught, when it should be introduced, and which factors support successful implementation. This broader perspective positions the present study as one of the most comprehensive syntheses currently available on AI curriculum development in medical education and provides a stronger evidence base for the design of longitudinal, competency-based, and outcome-oriented AI curricula capable of preparing future physicians for increasingly AI-enabled healthcare environments.

### 4.4 Limitations

The findings of this review should be interpreted within the context of the current developmental stage of AI curriculum integration in medical education. Despite the rapid expansion of AI-related educational initiatives, the literature remains characterized by substantial variation in curriculum design, competency definitions, instructional approaches, and evaluation strategies. This variability reflects an evolving field in which institutions are actively experimenting with different models of AI education, yet consensus regarding the optimal structure, scope, and sequencing of AI competencies within medical curricula has not been fully established. As a result, considerable diversity exists in how AI is conceptualized, taught, and assessed across educational settings.

A prominent limitation within the existing literature is the absence of standardized curricular frameworks that can guide AI competency development across the medical education continuum. While numerous studies emphasize the importance of AI literacy, ethical reasoning, critical appraisal, and human–AI collaboration, these competencies are frequently introduced through institution-specific initiatives with varying levels of curricular integration. Consequently, AI education often remains fragmented, ranging from isolated workshops and elective courses to integrated longitudinal experiences. This heterogeneity makes it difficult to determine which curricular approaches are most effective for fostering sustained competency development and preparing learners for AI-enabled clinical practice.

The current evidence base also demonstrates limited alignment between competency frameworks, learning activities, and assessment strategies. Many educational interventions describe what learners should know about AI, but fewer studies provide comprehensive curricular models that explicitly connect learning objectives, teaching methodologies, competency milestones, and evaluation mechanisms. From a curriculum development perspective, this represents a critical challenge because effective curricular integration requires coherence among educational outcomes, instructional design, and assessment practices. Without such alignment, it becomes difficult to determine whether learners are achieving the competencies required for responsible and effective use of AI in healthcare.

Another important limitation concerns the concentration of AI educational initiatives within specific stages of training. The literature demonstrates a predominance of preclinical and undergraduate interventions, whereas fewer studies have examined longitudinal integration extending into clinical clerkships, residency training, and continuing professional development. This distribution suggests that AI education is frequently introduced as an early educational topic rather than as a competency that develops progressively throughout professional formation. Given the increasing presence of AI technologies across healthcare systems, greater attention is needed to curricular models that support competency progression across the entire educational continuum, the rapid pace of technological innovation presents an ongoing challenge for curriculum development. AI capabilities, applications, and regulatory considerations continue to evolve, creating uncertainty regarding which competencies should be prioritized and how curricula can remain relevant over time. Traditional curriculum development processes may struggle to adapt at the same pace as technological change, emphasizing the need for flexible, competency-based curricular frameworks that can accommodate emerging innovations while maintaining core educational objectives.

## 5 Conclusion

The rapid integration of AI into healthcare is reshaping the competencies required of future physicians and increasing the need for curricular adaptation. This systematic review identified a broad range of relevant competencies, including AI literacy, critical appraisal, ethical reasoning, clinical decision-making, data interpretation, and human–AI collaboration. But the available evidence is largely descriptive, heterogeneous, and frequently based on self-reported outcomes, small samples, single-institution studies, and short follow-up periods.

Integrated, longitudinal, and active learning approaches were commonly reported across the included studies and were generally associated with positive educational outcomes. Nevertheless, the current evidence does not establish that these models are superior to alternative curricular approaches. Relatively, they should be considered promising strategies that warrant further evaluation through comparative, longitudinal, and methodologically robust studies using objective measures of competency development and clinical performance.

This review advances existing literature by providing a comprehensive curriculum-focused synthesis that integrates competency domains, curriculum models, pedagogical approaches, educational outcomes, faculty roles, learner engagement, and implementation facilitators within a single framework. The evidence supports the transition toward competency-based, longitudinal AI curricula capable of preparing future physicians to critically evaluate, ethically govern, and effectively collaborate with AI technologies. Such curricular transformation will be essential for ensuring that medical education remains aligned with the realities of modern healthcare and the evolving demands of clinical practice.

## Data Availability

The original contributions presented in the study are included in the article/supplementary material, further inquiries can be directed to the corresponding authors.
